# Cr_2_P_2_O_7_ as a Novel Anode Material for Sodium and Lithium Storage

**DOI:** 10.3390/ma13143139

**Published:** 2020-07-14

**Authors:** Shuo Wang, Tianyuan Zhu, Fei Chen, Xiang Ding, Qiao Hu, Jiaying Liao, Xiaodong He, Chunhua Chen

**Affiliations:** CAS Key Laboratory of Materials for Energy Conversions, Department of Materials Science and Engineering & Collaborative Innovation Center of Suzhou Nano Science and Technology, University of Science and Technology of China, Hefei 230026, China; ws14026@mail.ustc.edu.cn (S.W.); starzty@mail.ustc.edu.cn (T.Z.); chenf413@mail.ustc.edu.cn (F.C.); atp@mail.ustc.edu.cn (X.D.); huqiao@mail.ustc.edu.cn (Q.H.); ljy77@mail.ustc.edu.cn (J.L.); hxd427@mail.ustc.edu.cn (X.H.)

**Keywords:** chromium pyrophosphate, carbon coating, sodium ion battery, lithium ion battery

## Abstract

The development of new appropriate anode material with low cost is still main issue for sodium-ion batteries (SIBs) and lithium-ion batteries (LIBs). Here, Cr_2_P_2_O_7_ with an in-situ formed carbon layer has been fabricated through a facile solid-state method and its storage performance in SIBs and LIBs has been reported first. The Cr_2_P_2_O_7_@C delivers 238 mA h g^−1^ and 717 mA h g^−1^ at 0.05 A g^−1^ in SIBs and LIBs, respectively. A capacity of 194 mA h g^−1^ is achieved in SIBs after 300 cycles at 0.1 A g^−1^ with a high capacity retention of 92.4%. When tested in LIBs, 351 mA h g^−1^ is maintained after 600 cycles at 0.1 A g^−1^. The carbon coating layer improves the conductivity and reduces the side reaction during the electrochemical process, and hence improves the rate performance and enhances the cyclic stability.

## 1. Introduction:

Lithium ion batteries (LIBs) have been widely used for portable electronic devices and electric vehicles now [1,2]. The limited lithium resource and the uneven global distribution become the major issues for their larger-scale manufacture [3,4,5]. Sodium-ion batteries (SIBs) are promising alternative for LIBs due to sodium’s higher abundance, more even distribution and lower price compared to lithium [5]. For the materials choices, the larger ionic radius of Na^+^ versus Li^+^ (0.102 nm versus 0.076 nm) makes it impracticable to simply adopt most of the current anodes such as graphite for LIBs, exploration of appropriate anodes for SIBs is thus still necessary [6].

Nowadays the reported anode materials for SIBs mainly include carbon [7,8,9], alloys [10,11] and polyanion materials [12,13]. Among them, phosphate-based polyanionic materials have attracted enormous attention of the researchers due to their stable structural frameworks, in which there is a three-dimensional space for diffusion of the ions. In comparison with the metal oxides, transition metal polyanionic compounds usually demonstrate significant thermal stability as the strong covalent bonds between P and O can largely suppress the evolution of oxygen [5]. So far many polyanionic compounds such as FePO_4_ and VPO_4_ have been widely investigated as the anodes for LIBs [14,15,16,17,18,19,20]. However, quite few studies in literature have focused on the polyanionic compounds especially pyrophosphates as anode materials for SIBs. Wang et al. explored NaTi_2_(PO_4_)_3_@C which delivers a capacity of 208 mA h g^−1^ [21], while Hu et al. did a comparative study on layered TiP_2_O_7_ for Li/Na/K alkali metal batteries [22]. Fedotov et al. studied α-VPO_4_ as a novel anode material which delivers a capacity of 80 mA h g^−1^ for SIBs [23]. It is also noticed that, compared with other anode materials for SIBs, Cr_2_P_2_O_7_ shows advantages in materials cost as well as in electrochemical properties (Table 1).

Although the effect of pressure on the structural behavior of Cr_2_P_2_O_7_ was studied by Blanc et al. [27], it has never been investigated as an electrode material for batteries before. Considering the fact that phosphate-based polyanionic compounds always suffer from the poor electric conductivity which limits the electrochemical performance [28,29,30,31,32,33,34,35], it is necessary to apply a conducting carbon coating on the surface of the particles. For example, Kim et al. improved the rate capability and cycling stability of Na_3_V_2_(PO_4_)_3_ substantially through a carbon coating method [29]. The carbon coating can effectively improve the electrical conductivity, hence increasing the capacity and suppressing the capacity decay during cycling [36,37,38,39,40].

In this paper, we prepared the Cr_2_P_2_O_7_ through a facile solid-state reaction and first reported the electrochemical performances of Cr_2_P_2_O_7_ as anodes for LIBs and SIBs. In order to further investigate the influence of the surface modification, the carbon-coated sample Cr_2_P_2_O_7_@C was synthesized by in-situ forming a carbon layer on the particles, and its electrochemical performances in LIBs and SIBs were also measured. The Cr_2_P_2_O_7_@C shows a high capacity of 238 mA h g^−1^ at 0.05 A g^−1^ in SIBs. When measured in LIBs, it maintains 351 mA h g^−1^ after 600 cycles at 0.1 A g^−1^.

## 2. Experimental Section

### 2.1. Synthesis of Cr_2_P_2_O_7_ and Cr_2_P_2_O_7_@C

Stoichiometric ratio of chromium nitrate (Cr(NO_3_)_3_·9H_2_O) and diammonium hydrogen phosphate ((NH_4_)_2_HPO_4_) were dispersed in acetone and mixed by ball-milling for 10 h. The precursor was obtained after evaporating acetone at 110 °C in oven. Then it was sintered at 900 °C under argon atmosphere for 12 h to obtain Cr_2_P_2_O_7_. To synthesize the carbon coated samples, 10, 20 and 40 wt% polyvinylpyrrolidone (PVP) (relative to the total mass of Cr(NO_3_)_3_·9H_2_O and (NH_4_)_2_HPO_4_) were added respectively into the mixture before ball-milling. The synthesized carbon coated samples were named as Cr_2_P_2_O_7_-10PVP, Cr_2_P_2_O_7_-20PVP, Cr_2_P_2_O_7_-40PVP, respectively. The sample Cr_2_P_2_O_7_-20PVP was also named as Cr_2_P_2_O_7_@C.

### 2.2. Material Characterization

The phases and crystallinity of these products were characterized with an X-ray diffractometer (XRD, Rigaku TTR-III, Cu Kα radiation, Akishima, Tokyo, Japan. Their morphologies were observed by a scanning electron microscope (SEM, SIRION200, FEI, Hillsborough, OR, USA) or a transmission electron microscope (TEM, JEM-2100F, JEOL, Akishima, Tokyo, Japan). The elemental compositions were studied with energy-dispersive X-ray spectroscopy (EDS) equipped to the SEM instrument. Raman spectroscopy was performed by a Renishaw inVia Raman microscope (Gloucestershire, London, UK). The carbon content of Cr_2_P_2_O_7_@C was analyzed with an infrared carbon-sulfur analyzer (CS8800C, Jinbo, Wuxi, China). DC electrical conductivity was measured by a direct volt-ampere method (STR722, Jingge, Suzhou, China), in which disc samples pressed at 20 kPa were contacted with a four-point probe.

### 2.3. Electrochemical Measurements

The electrochemical performances of the samples were tested using coin-type half cell (CR2032, Kejing, Hefei, China). The working electrodes were prepared by mixing Cr_2_P_2_O_7_ or Cr_2_P_2_O_7_@C (70%), acetylene black (20%) and poly(vinylidene fluoride) (10%) binder in N-methyl-2-pyrrolidinone. The slurries obtained were uniformly coated on a copper foil collector, followed by vacuum drying. The mass loading of the active material in electrodes was about 1.5 mg cm^−2^. The lithium half cells were assembled in an argon-filled glovebox with lithium metal, Celgard 3501 polypropylene membrane (Celgard, Charlotte, NJ, USA) and 1 mol L^−1^ LiPF_6_ in EC/DMC (1:1, *v*/*v*) as the counter electrode, separator and electrolyte, respectively. For sodium half cells, sodium metal was used as counter electrode with Whatman glass fiber and 1 mol L^−1^ NaClO_4_ in EC/DMC (1:1, *v*/*v*) to which 1% fluoroethylene carbonate additive was added as the separator and electrolyte, respectively. The galvanostatic tests of the cells were conducted on a Neware BTS-610 multichannel battery test system (Neware Co., Shenzhen, China) in the voltage range of 0.1–3 V at room temperature (25 ± 2 °C). Cyclic voltammogram (CV) and electrochemical impedance spectroscopy (EIS) measurements were performed on a CHI660C electrochemical workstation (Chenhua Co., Shanghai, China). To measure the EIS spectrum, the batteries were activated at 50 mA g^−1^ for 2 cycles. The frequency range was from 10^5^ to 10^−2^ Hz with an applied AC amplitude of 5 mV. The open circuit voltage of the cell was about 2.0 V.

## 3. Results and Discussion

**Figure 1:**
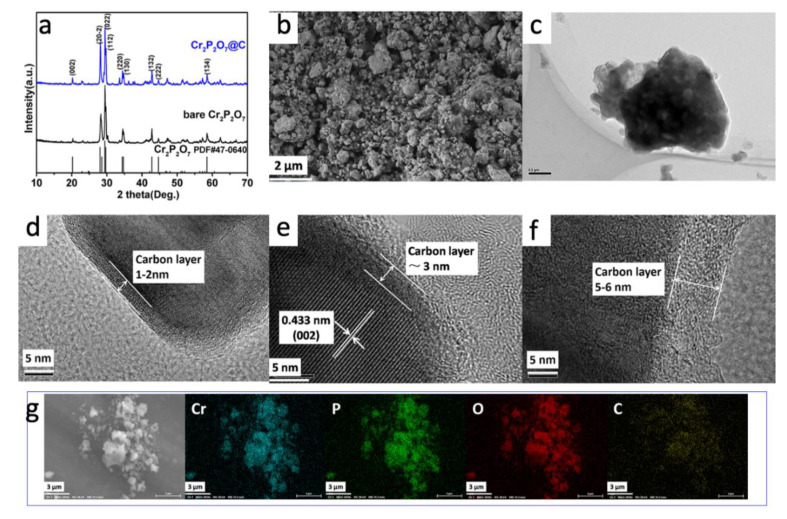
(**a**) XRD patterns of bare and carbon-coated Cr_2_P_2_O_7_ samples; (**b**) SEM image and (**c**) TEM image of Cr_2_P_2_O_7_@C. HRTEM images of (**d**) Cr_2_P_2_O_7_-10PVP, (**e**) Cr_2_P_2_O_7_-20PVP and (**f**) Cr_2_P_2_O_7_-40PVP. (**g**) EDS elemental mapping of Cr_2_P_2_O_7_@C.

**Figure 2:**
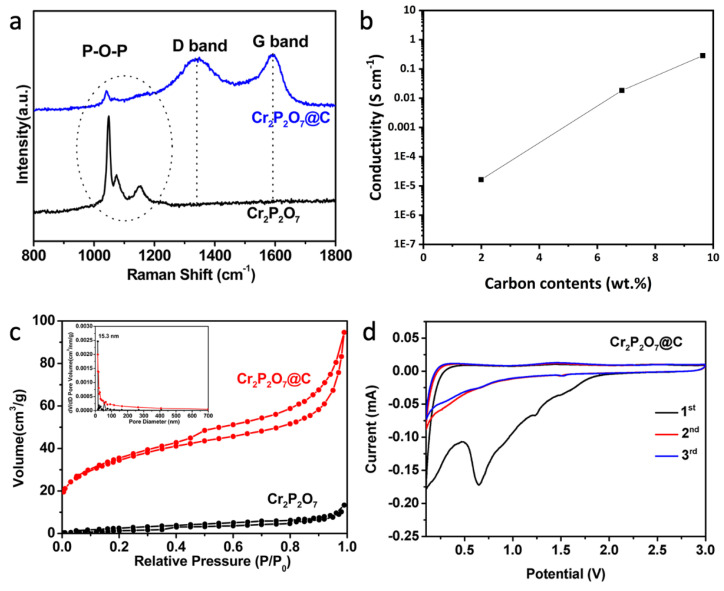
(**a**) Raman spectra of Cr_2_P_2_O_7_@C and Cr_2_P_2_O_7_; (**b**) Electronic conductivity of the carbon coated samples; (**c**) N_2_ adsorption-desorption isotherms and (inset) the pore size distribution curves of Cr_2_P_2_O_7_@C and Cr_2_P_2_O_7_; (**d**) CV curves in SIBs of Cr_2_P_2_O_7_@C in the first 3 cycles between 0 V and 3 V at a scanning rate of 0.1 mV s^−1^.

Herein, we synthesize a green powder Cr_2_P_2_O_7_ and the black carbon coated products through a facile solid-state reaction. All the peaks on their XRD patterns (Figure 1a) can be well indexed to the monoclinic structure (space group C2/c(15)), suggesting that both have high crystallinity and purity. Also, according to the SEM image of Cr_2_P_2_O_7_@C (Figure 1b), it has micron-sized secondary particles of irregular shape with a size less than 2 μm, which are agglomerated with small grains of 200–500 nm. While the bare sample shows a quite different morphology with an average particle size over 8 μm (Figure S1a, Supporting Information). There are numerous pores in the bulk since the gases escaped quickly during the heating process. The HRTEM image of Cr_2_P_2_O_7_@C (Figure 1e) shows the fine lattice fringes with a stripe distance of 0.433 nm, corresponding to the (002) plane of the monoclinic Cr_2_P_2_O_7_. The HRTEM image of the bare sample (Figure S1b) shows a lattice fringe of 0.303 nm which corresponds to the (022) planes. The EDS results of Cr_2_P_2_O_7_@C (Figure 1g) indicate a uniform distribution of the elements Cr, P, O and C. It further confirms the uniform carbon coating on the particles, which is of great benefit to the structural stability and the mitigation of the volume variation during cycling, therefore enhancing the electrochemical performance. The carbon coating layers with a thickness of 1–2, 3 and 5–6 nm can be observed clearly in Cr_2_P_2_O_7_-10PVP, Cr_2_P_2_O_7_-20PVP and Cr_2_P_2_O_7_-40PVP (Figure 1d–f), respectively. The carbon content of Cr_2_P_2_O_7_-10PVP, Cr_2_P_2_O_7_-20PVP and Cr_2_P_2_O_7_-40PVP analyzed by the infrared carbon-sulfur analyzer are 1.99 wt%, 6.85 wt% and 9.64 wt%, respectively. Furthermore, the Raman analysis confirms the presence of carbon (Figure 2a). Figure 2b shows the electronic conductivity of the carbon coated samples. The electronic conductivity of Cr_2_P_2_O_7_ is less than 5 × 10^−6^ S cm^−1^ and out of the measurement lower limit of the instrument. But for the carbon coated samples, they display orders of magnitude increase in the electronic conductivity. The nitrogen sorption analysis shows type-IV curves (Figure 2c), and the corresponding specific surface areas are 124.1 m^2^g^−1^ for Cr_2_P_2_O_7_@C and 7.0 m^2^g^−1^ for Cr_2_P_2_O_7_. The high surface area of Cr_2_P_2_O_7_@C should have advantages in the transportation of Na^+^ and the diffusion of electrolyte.

**Figure 3:**
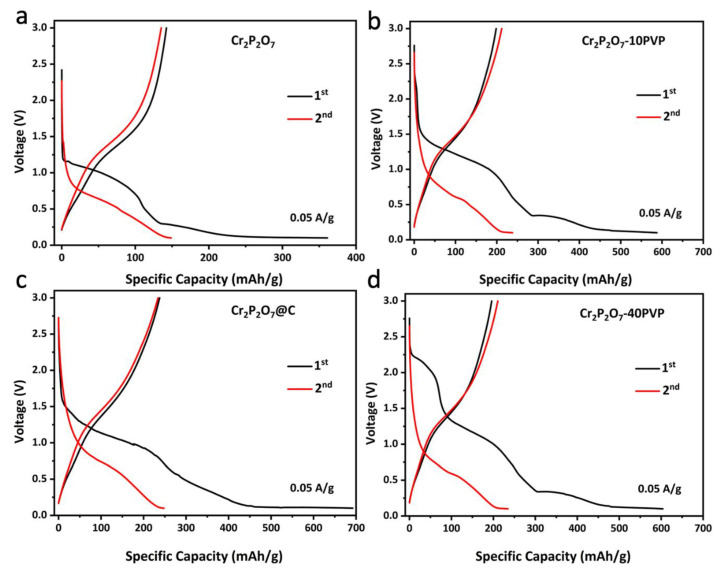
Charge-discharge profiles of (**a**) Cr_2_P_2_O_7_, (**b**) Cr_2_P_2_O_7_-10PVP, (**c**) Cr_2_P_2_O_7_-20PVP (Cr_2_P_2_O_7_@C) and (**d**) Cr_2_P_2_O_7_-40PVP for the initial 2 cycles in Na/Cr_2_P_2_O_7_ cells at 0.05 A g^−1^.

The sodium storage performances of the two samples are measured. From the CV curves of Cr_2_P_2_O_7_@C (Figure 2d), a sharp reduction peak below 0.2 V can be observed obviously in the initial cathodic process, corresponding to the formation of the solid electrolyte interface (SEI) layer. There are also two peaks at 0.7 V and 1.2 V, assigned to the conversion reaction. For the anodic process, a broad oxidation peak at 1.5 V is observed which can be assigned to backward conversion reaction. Furthermore, the curves of the subsequent cycles almost overlap with each other, suggesting the high stability and reversibility of Cr_2_P_2_O_7_@C, which are in contrast with the bare Cr_2_P_2_O_7_ (Figure S2). The discharge-charge profiles of Cr_2_P_2_O_7_@C (Figure 3c) are consistent with the CV results. The first-cycle charge capacity of Cr_2_P_2_O_7_-10PVP (Figure 3b), Cr_2_P_2_O_7_-20PVP and Cr_2_P_2_O_7_-40PVP (Figure 3d) reaches 199, 238 and 196 mA h g^−1^ at 0.05 A g^−1^, respectively. While it is only 142 mA h g^−1^ for bare Cr_2_P_2_O_7_ (Figure 3a). The increased capacity can be ascribed to the presence of the conductive carbon layer which can facilitate the conduction of electrons and help the sodium ions reach the inner cores of the particles. Obviously, Cr_2_P_2_O_7_-20PVP is the optimal sample.

**Figure 4:**
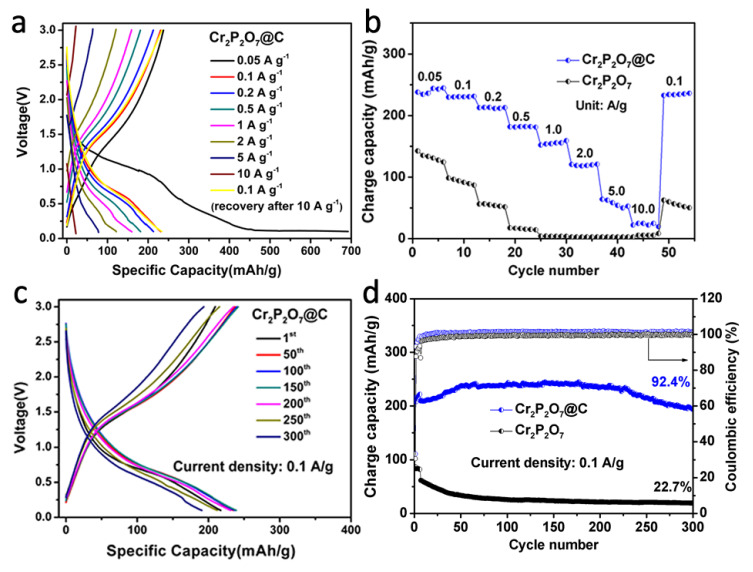
Electrochemical performance in Na/Cr_2_P_2_O_7_ cells: charge-discharge profiles of different current densities (**a**); comparison of the rate performances (**b**); charge-discharge curves of the selected cycles of Cr_2_P_2_O_7_@C at 0.1 A g^−1^ (**c**); comparison of the cycling performances (**d**). The cells were initially activated for three times at a low current density of 0.05 A g^−1^ and then cycled at 0.1 A g^−1^.

When increasing the current rate (Figure 4a), Cr_2_P_2_O_7_@C delivers excellent rate capacities of 230, 213, 182, 152, 121, 64, 22 mA h g^−1^ at different current densities of 0.1, 0.2, 0.5, 1.0, 2.0, 5.0, 10.0 A g^−1^, respectively. A capacity of 232 mA h g^−1^ can be achieved when returning to 0.1 A g^−1^ with no capacity decrease, indicating the high reversibility and good rate tolerance with the help of the in-situ formed carbon layer. As a comparison for the bare Cr_2_P_2_O_7_ (Figure 4b), lower capacities of 99, 55, 15 mA h g^−1^ are achieved at 0.1, 0.2, 0.5 A g^−1^, respectively, with ignorable capacity at higher current densities. When returning to 0.1 A g^−1^, it maintains only 63 mA h g^−1^ with a quite large capacity loss, suggesting a poor stability during the electrochemical process. As shown in Figure 4c,d, the cycling performances of the materials in Na/Cr_2_P_2_O_7_ cells were also tested. After 300 cycles at 0.1 A g^−1^, a capacity of 194 mA h g^−1^ is achieved with a high capacity retention of 92.4% for Cr_2_P_2_O_7_@C, while the bare Cr_2_P_2_O_7_ maintains a much lower capacity of 20 mA h g^−1^. After coated with a conductive carbon layer which can facilitate the conduction of electrons, Cr_2_P_2_O_7_@C also has smaller particle size with a microporous structure. The micro-porosity and higher specific area increase the contact area between material and electrolyte and shorten the path of ion diffusion. The carbon coating also reduces the direct contact between the material and the electrolyte and reducing the side reaction, which has a major impact on the improvement of cycle performance. And the volume expansion of the material can be alleviated to a certain extent. Therefore, the capacity and cyclability of the material are improved. When compared with other widely reported anode materials, the electrochemical properties of Cr_2_P_2_O_7_@C are also competitive (Table 1).

**Figure 5:**
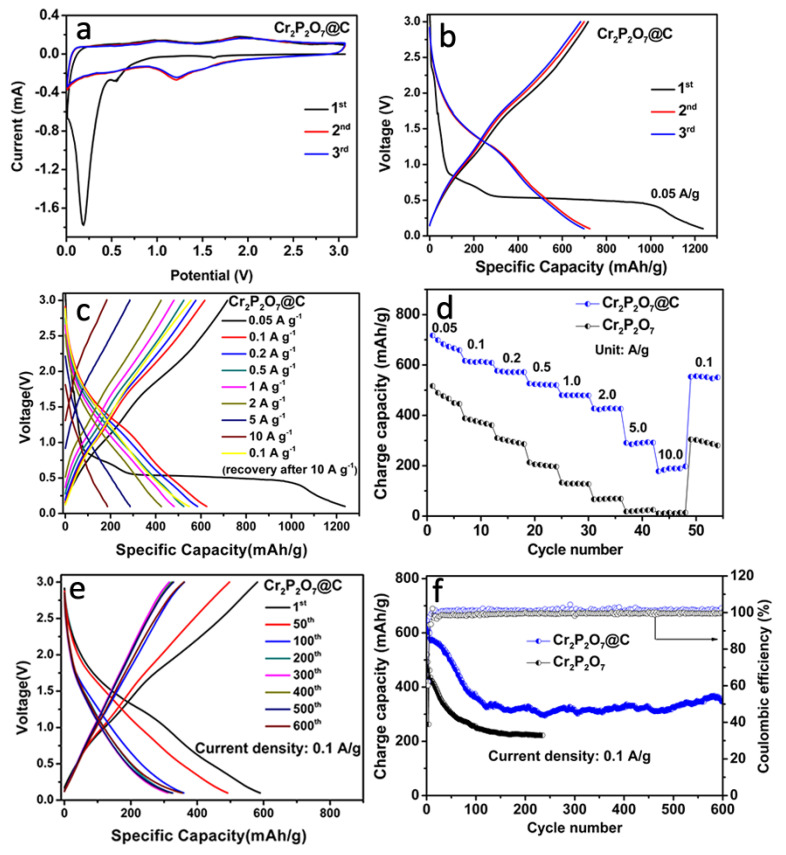
Electrochemical performance in Li/Cr_2_P_2_O_7_ cells: CV curves of Cr_2_P_2_O_7_@C in the first 3 cycles between 0 V and 3 V at a scanning rate of 0.1 mV s^−1^ (**a**); charge-discharge profiles for the initial 3 cycles (**b**); charge-discharge profiles of different current densities (**c**); comparison of the rate performances (**d**); charge-discharge curves of the selected cycles of Cr_2_P_2_O_7_@C at 0.1 A g^−1^ (**e**); comparison of the cycling performances (**f**). The cells were initially activated for three times at a low current density of 0.05 A g^−1^ and then cycled at 0.1 A g^−1^.

As a new anode material, the lithium storage performance of Cr_2_P_2_O_7_@C is also investigated. On its CV curves (Figure 5a), a sharp reduction peak at 0.2 V is observed which corresponds to the formation of the SEI layer in the initial cathodic process. There are also two minor peaks at 0.6 V and 1.7 V assigned to the conversion reaction. The anodic curves show two corresponding oxidation peaks at 1.0 V and 1.9 V. The CV curves of Cr_2_P_2_O_7_ show the same characteristics (Figure S3). On the charge-discharge voltage profiles of Cr_2_P_2_O_7_@C (Figure 5b), two plateaus at 0.6 V and 1.7 V in the first discharge and two plateaus at 1.0 V and 1.9 V in the charge profile are measured, which correspond exactly to the CV curves. Hence we propose following conversion reaction based on the charge/discharge profiles and the CV curves:

$$4A^{+}+4e^{-}+Cr_{2}P_{2}O_{7}\frac{\underline{Discharge}}{Charge}\;2Cr+A_{4}P_{2}O_{7}\;\left(A:Li,Na\right)$$

The initial charge capacity for Cr_2_P_2_O_7_@C in Li/Cr_2_P_2_O_7_ cells is 717 mA h g^−1^ at 0.05 A g^−1^, while it is only 517 mA h g^−1^ for Cr_2_P_2_O_7_ (Figure S4). The rate performance of Cr_2_P_2_O_7_@C is also markedly better than Cr_2_P_2_O_7_ (Figure 5c,d). The former shows capacities of 285 and 184 mA h g^−1^ at current densities of 5.0 and 10.0 A g^−1^, respectively, while the latter shows little capacity at these rates. It is further demonstrated that the in-situ fabricated carbon coating can effectively improve the electrochemical performance. For the cycling properties (Figure 5e,f), Cr_2_P_2_O_7_@C maintains a reversible capacity of 351 mA h g^−1^ after 600 cycles at 0.1 A g^−1^, which is much higher than that of the bare Cr_2_P_2_O_7_ with a capacity down to 223 mA h g^−1^ after 235 cycles. Note that the rapid decrease in the first dozen cycles of both samples may be due to the initial activation process.

**Figure 6:**
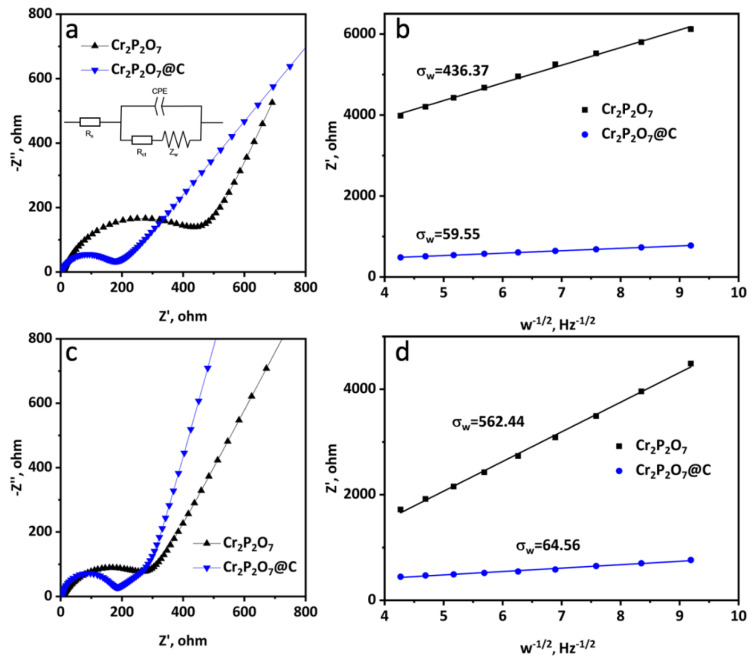
The fitting impedance spectra of Cr_2_P_2_O_7_@C and Cr_2_P_2_O_7_ in (**a**) Li/Cr_2_P_2_O_7_ and (**b**) Na/Cr_2_P_2_O_7_ cells. The equivalent circuit used for fitting the experimental EIS data of Li/Cr_2_P_2_O_7_ and Na/Cr_2_P_2_O_7_ cells (inset). Linear fitting to Z’ versus ω^−1/2^ plots in the low-frequency range in (**c**) Li/Cr_2_P_2_O_7_ and (**d**) Na/Cr_2_P_2_O_7_ cells.

The electrochemical impedance spectroscopy (EIS) test was measured in both Li/Cr_2_P_2_O_7_ and Na/Cr_2_P_2_O_7_ cells to study the electrochemical kinetics (Figure 6). Cr_2_P_2_O_7_@C exhibits lower charge transfer resistance and electrolyte resistance than the bare Cr_2_P_2_O_7_ in both cases (Table 2 and Table 3). Due to the incorporation of the carbon layer, the calculated apparent lithium diffusion coefficient and apparent sodium diffusion coefficient of Cr_2_P_2_O_7_@C are 2.7 × 10^−12^ cm^2^ s^−1^ and 2.3 × 10^−12^ cm^2^ s^−1^, respectively, which are higher than those of Cr_2_P_2_O_7_ (5.1 × 10^−14^ cm^2^ s^−1^ and 3.0 × 10^−14^ cm^2^ s^−1^). Such a huge difference in apparent diffusion coefficient is mainly due to the difference in specific area (124.1 m^2^g^−1^ for Cr_2_P_2_O_7_@C vs. 7.0 m^2^g^−1^ for Cr_2_P_2_O_7_) because the area used to calculate the diffusion coefficient is the geometric area which is much less than the real contact area between the electrode and the liquid electrolyte. Thus the in-situ carbon coating method efficiently improves the cycle and rate performances of lithium and sodium cells.

## 4. Conclusions

To explore new anode materials for LIBs and SIBs, a pyrophosphate powder, i.e., Cr_2_P_2_O_7_, is synthesized via a facile solid-state reaction method. An in-situ carbon coating strategy is also taken to enhance its electronic conductivity and thus the apparent alkali ion diffusion coefficient. Such an anode material exhibits good electrochemical properties both in rate and cycling performances in LIBs and SIBs. The in-situ carbon layer is very important for the improvement compared to the non-coated samples. This novel phosphate-based anode material is also expected to promote the overall safety of the batteries.

## Figures and Tables

**Table 1:** Cost and electrochemical properties (for SIBs) comparison of Cr_2_P_2_O_7_ with other anode materials.

Anode Material	Raw Material	Raw Cost * (¥ kg^−1^)	Cycle Capability (mA h g^−1^)	Capacity (mA h g^−1^)	Ref.
Cr_2_P_2_O_7_@C	Cr(NO_3_)_3_·9H_2_O and Diammonium hydrogen phosphate	385	194 (300 cycles)	230 (100 mA g^−1^)	This work
Na_4_Ti_5_O_12_/C	Sodium carbonate and Tetrabutyl titanate	680	81 (300 cycles)	92 (100 mA g^−1^)	[24]
Mg_0.5_Ti_2_(PO_4_)_3_@C	Magnesium acetate, Diammonium hydrogen phosphate and Titanium isopropoxide	520	130 (300 cycles)	200 (100 mA g^−1^)	[25]
NaTi_2_(PO_4_)_3_/C	Sodium acetate, Tetrabutyl titanate and Phosphoric acid	609	170 (500 cycles)	208 (100 mA g^−1^)	[21]
MoS_2_/C	Sodium molybdate dehydrate and Thiourea	758	352 (200 cycles)	400 (100 mA g^−1^)	[26]

* The costs are calculated based on the Aladin prices of the raw materials.

**Table 2:** Results of electrochemical impedance and Warburg coefficient of Cr_2_P_2_O_7_ in Li/Cr_2_P_2_O_7_ cell.

Samples	R_s_, Ω	R_ct_, Ω	σ_w_, Ω s^−1^	$D_{Na^{+}}$, cm^2^ s^−1^
Cr_2_P_2_O_7_	10.5	466.2	436.4	5.1 × 10^−14^
Cr_2_P_2_O_7_@C	5.7	131.6	59.6	2.7 × 10^−12^

**Table 3:** Results of electrochemical impedance and Warburg coefficient of of Cr_2_P_2_O_7_ in Na/Cr_2_P_2_O_7_ cell.

Samples	R_s_, Ω	R_ct_, Ω	σ_w_, Ω s^−1^	$D_{Li^{+}}$, cm^2^ s^−1^
Cr_2_P_2_O_7_	8.6	303.7	562.4	3.0 × 10^−14^
Cr_2_P_2_O_7_@C	5.3	159.2	64.6	2.3 × 10^−12^

## Supplementary Materials

The following are available online at https://www.mdpi.com/1996-1944/13/14/3139/s1, Figure S1. SEM image (a) and TEM image (b) of Cr_2_P_2_O_7_. Figure S2. The CV curves of Cr_2_P_2_O_7_ in the first 3 cycles between 0 V and 3 V at a scanning rate of 0.1 mV s^−1^ in SIBs. Figure S3. The CV curves of Cr_2_P_2_O_7_ in the first 3 cycles between 0 V and 3 V at a scanning rate of 0.1 mV s^−1^ in LIBs. Figure S4. The charge-discharge profiles of Cr_2_P_2_O_7_ for the initial 3 cycles in LIBs.
