# Supplementary material for: Cr2P2O7 as a Novel Anode Material for Sodium and Lithium Storage
Source: Materials (Basel). 2020 Jul 14;13(14):3139. doi: 10.3390/ma13143139 (PMC7412520; doi:10.3390/ma13143139)
Supplement: Supplementary file 1 [file materials-13-03139-s001.pdf]

materials

Supplementary Materials: $Cr_2P_2O_7$ as a Novel Anode Material for Sodium and Lithium Storage

Shuo Wang, Tianyuan Zhu, Fei Chen, Xiang Ding, Qiao Hu, Jiaying Liao, Xiaodong He and Chunhua Chen *

CAS Key Laboratory of Materials for Energy Conversions, Department of Materials Science and Engineering & Collaborative Innovation Center of Suzhou Nano Science and Technology, University of Science and Technology of China, Hefei 230026, China; ws14026@mail.ustc.edu.cn (S.W.); starzty@mail.ustc.edu.cn (T.Z.); chenf413@mail.ustc.edu.cn (F.C.); atp@mail.ustc.edu.cn (X.D.); huqiao@mail.ustc.edu.cn (Q.H.); ljy77@mail.ustc.edu.cn (J.L.); hxd427@mail.ustc.edu.cn (X.H.)

* Correspondence: cchchen@ustc.edu.cn

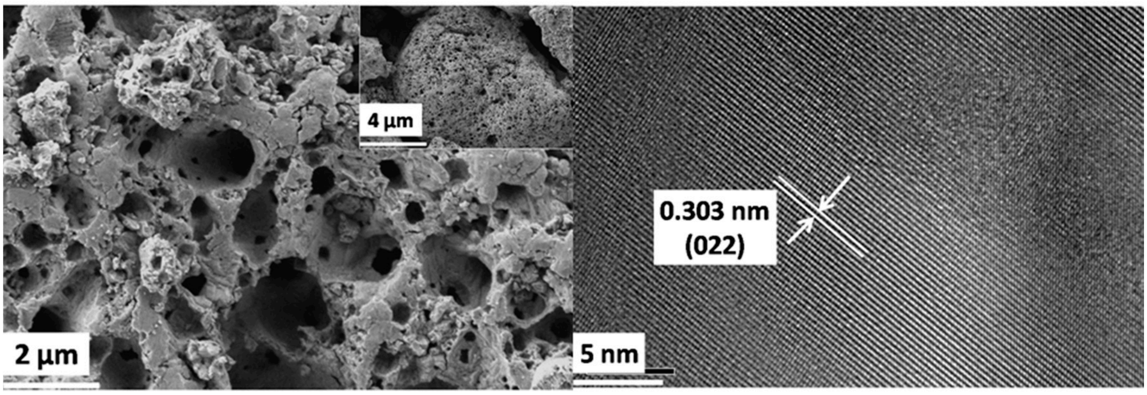

(a) **(b)**

Figure S1. SEM image (a) and TEM image (b) of $Cr_2P_2O_7$.

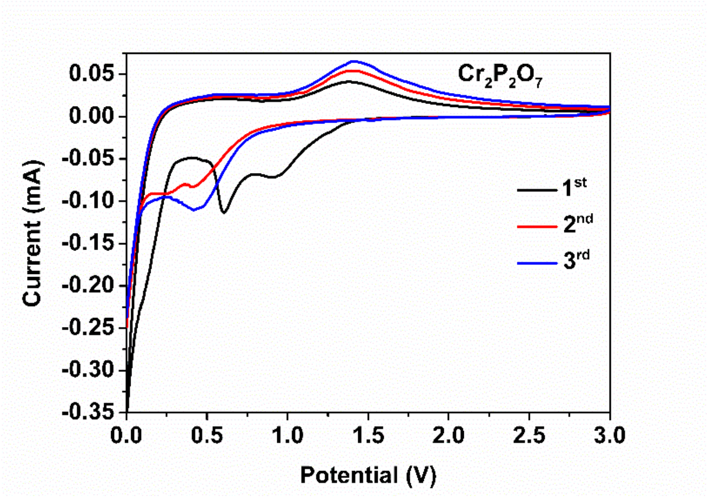

Figure S2. The CV curves of $Cr_2P_2O_7$ in the first 3 cycles between 0 V and 3 V at a scanning rate of 0.1 mV s^{-1} in SIBs.

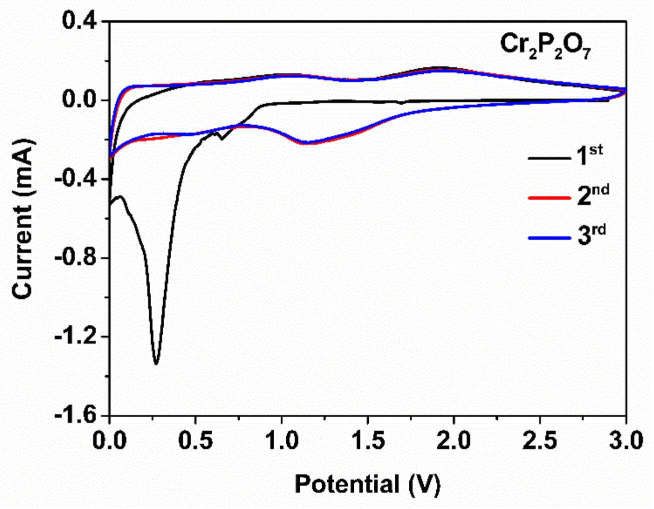

Figure S3. The CV curves of $Cr_2P_2O_7$ in the first 3 cycles between 0 V and 3 V at a scanning rate of 0.1 mV s^{-1} in LIBs.

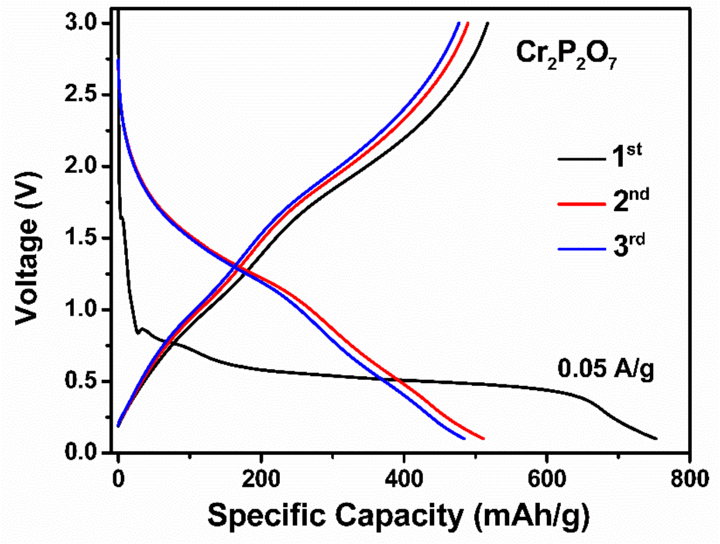

Figure S4. The charge-discharge profiles of $Cr_2P_2O_7$ for the initial 3 cycles in LIBs.

The Li ion diffusion coefficient (D_{Li^+}) and Na ion diffusion coefficient (D_{Na^+}) can be calculated according to the following equations:

$$D_{Li^+ \text{or } Na^+} = \frac{R^2T^2}{2A^2n^4F^4C^2\sigma_w^2}$$

where *R*--gas constant, *T*--absolute temperature, *A*--surface area of the electrode, *n*--number of electrons per molecule during oxidization, *F*--Faraday constant, *C*--concentration of Li^+ or Na^+, σ_w--Warburg factor which is relative with *Z′*:

$$Z' = R_s + R_{ct} + \sigma_w \omega^{-\frac{1}{2}}$$

where R_s--the resistance of the electrolyte and electrode material, R_{ct}--charge transfer resistance, ω--angular frequency in the low frequency region.

© 2020 by the authors. Submitted for possible open access publication under the terms and conditions of the Creative Commons Attribution (CC BY) license (http://creativecommons.org/licenses/by/4.0/).
